# Serum erythropoietin in acute ischemic stroke: preliminary findings

**DOI:** 10.1038/s41598-024-53180-3

**Published:** 2024-02-01

**Authors:** Lisda Amalia, Gilang Nispu Saputra

**Affiliations:** Department of Neurology, Faculty of Medicine, Hasan Sadikin Hospital, Universitas Padjadjaran, Jl. Eykman 38, Bandung, 40161 Indonesia

**Keywords:** Cell biology, Neuroscience, Neurology

## Abstract

Ischemic stroke is the most common stroke, caused by occlusion of cerebral vessels and leading causes of disability. Erythropoietin (EPO) has non-hematopoietic effects as a neuroprotectant after ischemic event. This study aimed to learn the serum level of EPO in acute ischemic stroke. This cross-sectional study of ischemic stroke patients with onset < 24 h and consecutive sampling was used to collect the data from medical records review, physical examinations, head CT, 24-h EPO, 24-h and seventh-day NIHSS. A total of 47 patients consisting of 59.6% women, with a median age of 53 years old (21–70). The median 24 h EPO level was 808.6 pg/mL (134.2–2988.9). The relationship between 24 h-EPO and 24-h NIHSS were not significant (r = 0.101; *p* = 0.250), nor to 7th day NIHSS (r =  − 0.0174; *p* = 0.121) and to delta NIHSS (r = 0.186; *p* = 0.106). The relationship of blood collection time (hour) and EPO was significant (r =  − 0.260; *p* = 0.039). There was a statistically significant difference between serum EPO levels in ischemic stroke patients with lacunar stroke compared to non-lacunar stroke (288.5 vs. 855.4 ng/mL; *p* = 0.021). There was a relationship between the time of collection of blood and the level of EPO and also there was difference EPO level in lacunar stroke subtype compared with non-lacunar. The relationship between EPO and NIHSS lost significance after analysis. There is a need for a future study comparing each stroke risk factor and the same blood collection time.

## Introduction

Stroke is one of the most common causes of death^1^. The incidence of stroke will increase as much as 12% in developed countries and 20% in developing countries^2^. Data from The Indonesian Ministry of Health in 2018 showed the prevalence of stroke was 10@.9/1000, increasing from 7/1000 in 2013^3^. Stroke is an acute condition related decrease to abrupt in causes disturbance or loss of neurological function for more than 24 h^4^. Ischemic stroke accounts for 87% of all stroke cases related to cerebral vessel occlusion^1^.

Decreasing cerebral blood flow is accompanied by a decrease in cerebral perfusion and led to an ischemic condition with a necrotic core and penumbra area surrounding it. There will be bioenergy failure in the necrotic core resulting in cell death, while the penumbra area carries out self-rescue with ischemic preconditioning. A disproportion in the supply and oxygen demand in cerebral tissue promotes a sequence of biochemical and molecular events that leads to neuron cell death. Following a hypoxia condition, brain responses in many ways. One of the most intriguing ways is by producing preconditioning hypoxia protein which includes transcription factors Hypoxia-inducible factor-1α (HIF-1α) in the penumbra area, and induce EPO and Vascular Endothelial Growth Factors (VEGF) a as a self-protect mechanism within minutes of the ischemic process and reaches its peak within 24 h of the ischemic stroke^5–9^. Therefore, a deeper understanding regarding the role of EPO, specifically in ischemic stroke, is required to improve recovery and brain repair process after stroke.

## Methods

### Study design and population

This cross-sectional study of ischemic stroke patients with onset < 24 h and consecutive sampling was used to collect the data from medical records review, physical examinations, head CT, 24-h EPO, 24-h and seventh-day NIHSS.

### Clinical and imaging evaluation

The research subjects were patients diagnosed with ischemic stroke confirmed by CT scan (expertised by radiologist and confirmed by stroke neurologist) who came to the hospital within 24 h after stroke onset and were grouped into non-lacunar stroke and lacunar stroke. Inclusion criteria were a clinical diagnosis on the admission of < 24 h, aged 18–70 years old and have not got intravenous or oral drugs. We excluded patients with acute myocardial infarction, acute kidney injury, acute limb ischemia, dementia, respiratory failure, head trauma, history of malignancies, hematology disorders, hypoglycemia, history of surgery in the last three months, congestive heart failure, atrial fibrillation, liver dysfunction, significant of ischemic cerebral, a stroke of unknown onset, and hospital-acquired pneumonia. The results of the NIHSS criteria assessment are scores, namely a score of < 4 indicating a mild neurological deficit, a score of 5–12 indicating a moderate neurological deficit, and a score of > 12 indicating a severe neurological deficit^10^.

### EPO measurement

Examination of serum EPO levels was on admission using Enzyme Link Immunosorbent Assay (ELISA). The serum formed was separated and put into a microtube and then stored at − 80 °C until a predetermined number of samples was obtained. Serum EPO (pg/mL) was determined using a human EPO Elisa kit (E-EL-H3640, Elabscience, USA).

### Statistics

Pearson and spearman were used to see the correlation between 24-h onset EPO and NIHSS (24 h and seventh-day NIHSS). The t-Test and Mann Whitney U tests were used to compare EPO level in category severity of stroke. A *p*-value of less than 0.05 was considered statistically significant.

### Ethical approval

Dr. Hasan Sadikin General Hospital Bandung Human Research Ethics Committee (LB.02.01/X/6.5.251.2019) had approved this study. This study had complied with all relevant ethical regulations (including The Declaration of Helsinki).

## Result

A total of 124 subject with ischemic stroke was enrolled from August to December 2019, subjects were excluded due to other diseases, 11 subjects with > 24 h onset, five deaths, and nine subjects with hospital-acquired pneumonia. In the end, we recruited 47 patients (see supplementary material). Of the total subjects, 59.6% were women and, the median age was 53 (21–70) years. The level of hemoglobin, leukocyte, platelet, erythrocyte was normal compared to the reference value. Risk factor included hypertension (74.5%), hyperlipidemia (74.5%), diabetes mellitus (25.5%), hyperuricemia (10.6%) (Table 1).

**Table 1:** Characteristic of stroke patients.

Variable			n = 47
		n (%)	Mean ± SD or Median (Min–Max)
Sex			
Male		19 (40.4)	
Women		28 (59.6)	
Age (years)			53 (21–70)**
Laboratory test			
Hemoglobin (g/dL)			14.8 (10.5–16.5)**
Hematocrite (%)			43.4 ± 4.3*
Leukocyte (per μL)			9300 (6050–15,800)**
Erythrocyte (10^6^/μL)			5.1 (4.1–5.8)**
Platelet (per μL)			261,000 (206,000–538,000)**
Random Glucose (mg/dL)			136 (112–299)**
Total Cholesterol (mg/dL)			205 ± 44*
HDL (mg/dL)			41 (24–65)**
LDL (mg/dL)			65 (19–210)**
Triglyceride /TG (mg/dL)			142 ± 45*
Fasting Glucose (mg/dL)			124 (86–278)**
Post Prandial Glucose (mg/dL)			145 (92–362)**
Uric Acid (mg/dL)			5.7 (2.6–13.9)**
HbA1C (%)			6.5 ± 2.3*
Risk Factor			
Multiple		37 (78.7)	
Single		10 (21.3)	
Risk Factor			
Hypertension		35 (74.5)	
Diabetes Melitus		12 (25.5)	
Hyperlipidemia		35 (74.5)	
EPO (pg/mL)		808.6	(134.2–2988.9)**
Blood Collect Time (Hour)		12 (6–18) **	
NIHSS 24-h		6 (3–13)**	
EPO level based on stroke subtype			
Lacunar stroke	11 (23.4)	288.5 (134.2–495.2)	
Non-lacunar stroke	36 (76.6)	855.4 (466.7–2988.9)	
Category NIHSS 24-h			
Mild Stroke	11 (23.4)		
Moderate Stroke	36 (76.6)		
NIHSS 7th day		2 (1–6)**	
Category NIHSS 7th day			
Mild Stroke	40 (85.1)		
Moderate Stroke	7 (14.9)		
Delta NIHSS		4 (0–7)**	

Data are number (%) or mean (standard deviation) or median (interquartile range). *normal distribution data presented with mean (SD), **non normal distribution data presented with median.

This study showed median EPO 24 h 808.6 pg/mL, with a median 24 h NIHSS of 6 and 76.6% accounted for moderate stroke. The median of the seventh day NIHSS was seventh and, 85.1% accounted for a mild stroke. The median of delta NIHSS was 4, with 4.3% subject without changing NIHSS (Table 1).

This study also showed us that there was no significant relationship between comorbidities and other basic characteristics in lacunar and non-lacunar groups (*p* > 0.05) except increasing of total cholesterol and hypertrigliseride (Table 2).

**Table 2:** Characteristic of stroke patients based on stroke subtype.

Variable		Stroke subtype	
	Lacunar groups (n = 11)	Non lacunar groups (n = 36)	*p*-value
Sex			0.205
Male	10 (52.6)	9 (47.3)	
Women	14 (50)	14(50)	
Age (years)	65 (60–71 )	53 (21–60)	0.190
Laboratory test			
Hemoglobin (g/dL)	14.7 (12.5–15)	14.8 (10.5–16.5)**	0.257
Hematocrite (%)	43.2 ± 4.0	43.4 ± 4.3*	0.391
Leukocyte (per μL)	8900 (7000–10.500)	9300 (6050–15,800)**	0.211
Erythrocyte (10^6^/μL)	5.3 (4.5–5.5)	5.1 (4.1–5.8)**	0.178
Platelet (per μL)	250,000 (190,000–350,000)	261,000 (206,000–538,000)**	0.102
Random Glucose (mg/dL)	138 (100–245)	136 (112–299)**	0.166
Total Cholesterol (mg/dL)	200 ± 45	205 ± 44*	0.028
HDL (mg/dL)	40 (25–60)	41 (24–65)**	0.387
LDL (mg/dL)	70 (25–220)	65 (19–210)**	0.127
Triglyceride /TG (mg/dL)	140 ± 40	142 ± 45*	0.021
Fasting Glucose (mg/dL)	120 (90–250)	124 (86–278)**	0.479
Post Prandial Glucose (mg/dL)	150 (95–280)	145 (92–362)**	0.478
Uric Acid (mg/dL)	5.3 (2.8–11.0)	5.7 (2.6–13.9)**	0.169
HbA1C (%)	6.7 ± 1.9	6.5 ± 2.3*	0.197
Risk factors			
Hypertension	20 (57.1)	15 (42.8)	0.240
Diabetes Mellitus	6 (50)	6 (50)	0.108
Hyperlipidemia	20 (57.1)	15 (42.8)	0.151
Hyperuricemia	23 (51.1)	22 (48.8)	0.182

Data are number (%) or mean (standard deviation) or median (interquartile range). *normal distribution data presented with mean (SD), **non normal distribution data presented with median.

**Figure 1:**
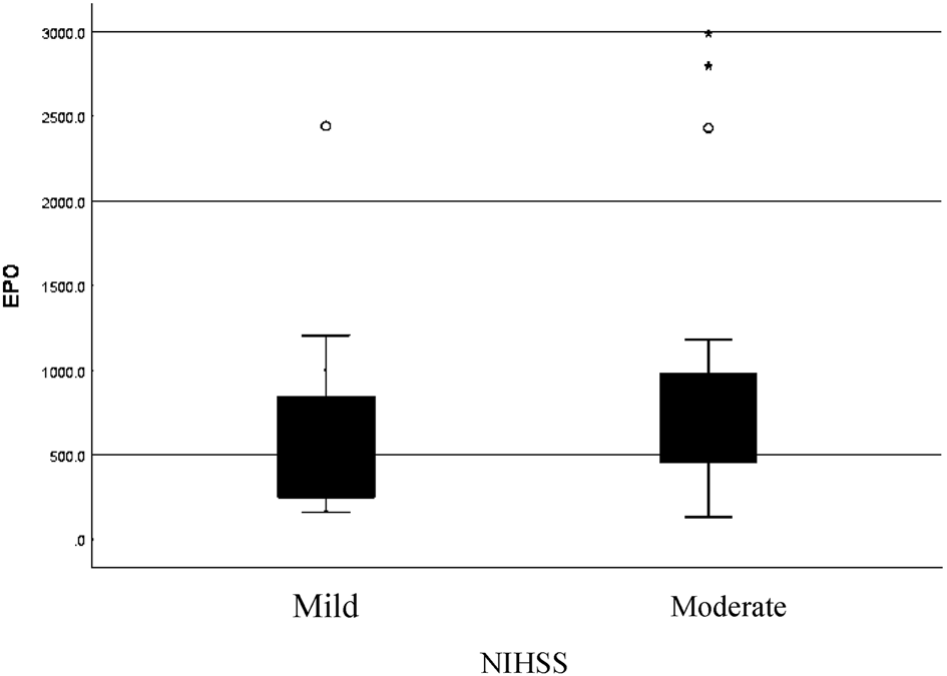
Boxplot 24-h EPO and 24-h NIHSS.

This study showed a positive trend between 24-h EPO and 24-h NIHSS (r = 0.101 , *p* = 0.250), negative trend between 24-h EPO and 7th day NIHSS (r =  − 0.174, *p* = 0.121) and positive trend between 24-h EPO and delta NIHSS (r = 0.186, *p* = 0.106) (Table 3 and Fig. 1).

**Table 3:** Correlation of EPO and NIHSS.

Variable	Coeffecient of correlation (r)	IK 95%	*p* score
24-h EPO–24 h NIHSS	0.101	− 0.190–0.374	0.250
24-h EPO–7th day NIHSS	− 0.174	− 0.435–0.118	0.121
24-h EPO–Delta NIHSS	0.186	− 0.106–0.445	0.106

Rank spearman was used for analysis.

Data analysis showed us that in 24-h, median EPO in mild stroke was 705.5 (159.9–2441.8) pg/mL and in moderate stroke was 822.5 (134.2–2988.9) pg/mL, but statistically, there was no significant difference. There was a statistically significant difference between serum EPO levels in ischemic stroke patients with lacunar stroke compared to nonlacunar stroke ( 288.5 vs. 855.4 ng/mL; *p* = 0.021) (Table 4).

**Table 4:** Difference between serum EPO in *lacunar stroke* and* nonlacunar stroke group.*

Stroke subtype	EPO level (pg/mL)		*p*-value*
	Median	Minimum–Maximum	
Lacunar stroke (n = 11)	288.5	(134.2–495.2)	0.021
Nonlacunar stroke (n = 36)	855.4	(466.7–2988.9)	

*Mann Whitney Test (*p* < 0.05).

**Figure 2:**
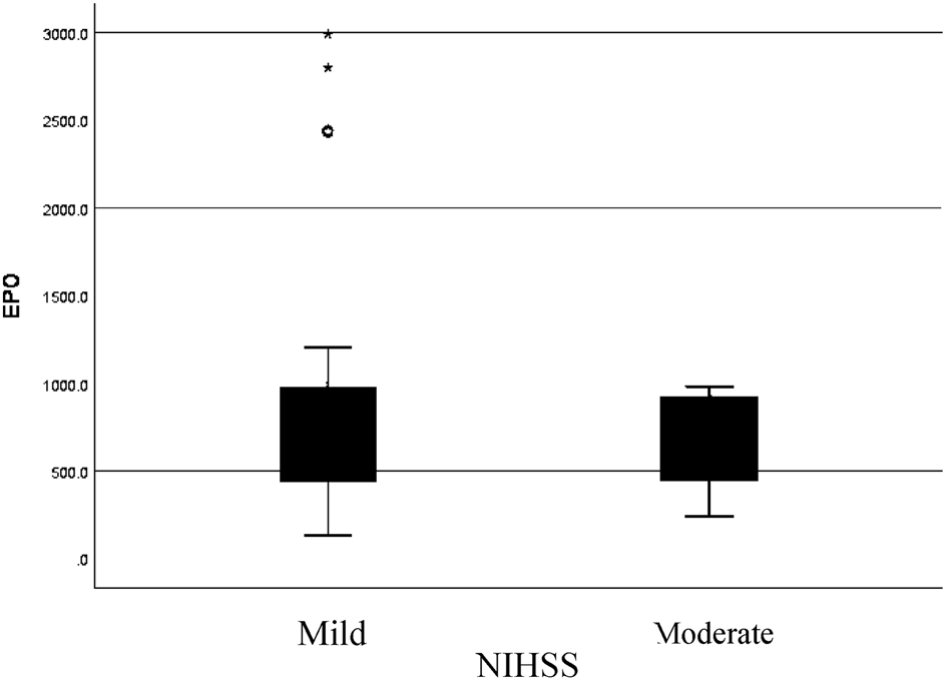
Boxplot 24-h EPO and 7th day NIHSS.

Data analysis showed us in seventh day, median EPO in mild stroke was 814.6 (134.2–2988.9) pg/mL and in moderate stroke was 428.3 (239.5–980) pg/mL, and statistically, there was no significant differentiation (Table 3 and Fig. 2).

**Figure 3:**
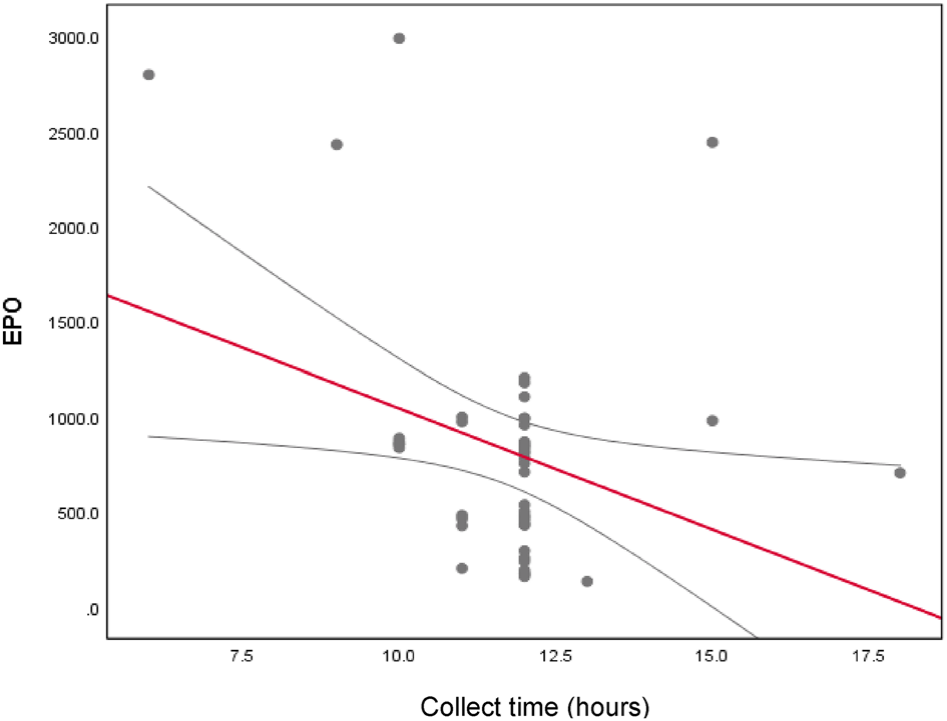
Scatterplot blood collect time and EPO.

The median blood collection time in this study was 12 (6–18) hours; data analysis showed a significant correlation between time to collect and EPO level, with a weak correlation and negative trend ( r = -0.260; *p* = 0.039) (Table 3 and Fig. 3).

## Discussion

A total of 47 subjects was included in this study, acute ischemic stroke patients with 59.6% of women with the median age of 53 years. This data was similar with a characteristic patient of Sahlgrenska Academy Study on Ischemic Stroke (SAHLSIS) with a mean of 56 years. Hypertension and hyperlipidemia were the most common risk factor in this study, with hypertension could be a single risk factor and the most common risk factor for stroke^11,12^.

There was a positive trend of the relationship between 24 h EPO and delta NIHSS, but also statistically was not significant (r = 0.186, *p* = 0.106). This finding is consistent with a previous study from SAHLSIS that showed a negative relationship between 24 h EPO and Scandinavian Stroke Scale (SSS), but not significant statistically as well^11^.

Data analysis showed there was a negative correlation between blood collect time (hour) and EPO ( r =  − 0.260; *p* = 0.039). There was no requirement time for exact blood collection time for this study, so the result for EPO might also be influenced. SAHLSIS study showed there is no significant differentiation between time (day) and EPO serum results^12^.

EPO serum could decrease in these situations such as chronic anemia, liver dysfunction, elderly, dementia but increase in hypoglycemia, head trauma or surgery history, hematologic disease, malignancy, atrial fibrillation, congestive heart failure. All of the mentioned were exclusion criteria in this study, but retinal disease, hyperglycemia, hyperlipidemia were not excluded^13–17^.

From this study results showed that there was no significant relationship between comorbidities and other basic characteristics in lacunar and non-lacunar groups (*p* > 0.05) except increasing of total cholesterol and hypertrigliseride. Some studies showed that EPO induces lipid catabolism activation via JAK2-STAT5 in adipose tissue, and later it produces triglycerides lipase (ATGL) so that decreased triglycerides serum level is expected, but there was no explanation on the relationship between cholesterol to EPO, according to this study there was a significant relationship between total cholesterol or triglyceride and EPO^18^. According to previously published results in chronic kidney disease patients, such inflammatory mediators as lipopolysaccharide and cytokines inhibit cholesterol efflux from cells by decreasing expression of the adenosine triphosphate–binding cassette A1 (ABCA1) gene. This effect leads to the decrease in reverse cholesterol transport and consequently the decrease in serum HDL-C levels. Furthermore, it was suggested that EPO expresses anti-inflammatory activities^19^. Further research is needed for investigating correlation between EPO level and lipid metabolism in acute ischemic stroke patients.

Sex in this study was dominated by women (59.6%). In previous study showed that disability and quality of life in stroke women would be worse than men. That could be a bias factor for increasing NIHSS. Men will also have a 10% higher differentiation erythrocyte mass index than women related to hormonal factors, iron needs due to menstruation, pregnancy, and lactation^20^.

A total of 74.5% of subjects in this study had hypertension and, 25.5% with diabetes mellitus. This condition is related to chronic inflammation, which would induce immune activation such as T cell and monocyte which secreted pro-inflammatory cytokine interferon-gamma (IFN). This pro-inflammatory cytokine will influence the metabolism of iron in the bone marrow and decrease the sensitivity of EPO. On the other hand, this cytokine will increase the severity level of stroke and contribute to some bias for this study^18,21–24^.

This study showed that the median level of 24-h EPO 808.6 pg/mL was higher than the SAHLSIS study (in Sweden population) with a mean of 9.3mIU/mL. There was a statistically significant difference between serum EPO levels in ischemic stroke patients with lacunar stroke compared to non-lacunar stroke (288.5 vs. 855.4 ng/mL; *p* = 0.021). Small lesions in lacunar stroke will certainly produce small ischemic lesions, this will result in a better clinical outcome in lacunar stroke than non-lacunar^25^. A wide brain damage also correlates with worsening of neurological deficit in acute ischemic stroke patients and wide ischemic changes were found in the non-lacunar group. This finding showed the extent of neuronal brain damage after ischemic stroke^5^. It should be borne in mind that lacunar infarcts are the stroke subtype with the best functional prognosis even in pure motor stroke, which is the lacunar syndrome with the greatest functional impairment, and we can asess using NIHSS on admission and discharge^24^.

Tissue hypoxia and cerebral ischemia activate HIF-1α, which in turn activates transcription of the EPO and Vascular Endothelial Growth Factor (VEGF) genes. HIF-1α, EPO, and VEGF increased as early as 1 h after acute ischemic stroke and may be used as marker of severity of neuronal damage and recovery in brain ischemic. There were strong correlations between HIF-1α and on admission and discharge NIHSS^5^. The main targets of EPO are neurons, while VEGF prevents apoptosis and induces endothelial cell proliferation, resulting in angiogenesis and improved tissue oxygenation^5,26^. EPO also contributes to endothelial cell proliferation, and VEGF also has a direct neuroprotective effect on neuronal cells. EPO and VEGF receptors are also generated on microglial cells and astrocytes, the targets of which are glial cells, but the effect on neuronal survival is unclear. EPO is thought to be able to stop the signal for cell death thereby reducing infarct volume^14^. There is a need to study further the possible influence of environmental and gene interactions with EPO levels in acute ischemic stroke^24,27,28^. Due to stroke, HIF-1α and EPO starts to accumulate as a response to inadequate oxygen level. HIF-1α mRNA expression and EPO after hypoxia could be detected within first thirty to sixty minutes. The regulation of HIF-1α and EPO, in penumbra, is rising until 7.5 h after onset of ischemia, so blood collection time (less then 7.5 h onset) could influence the EPO level in acute ischemic stroke^5^. Therefore, a deeper understanding regarding the role of EPO, specifically in ischemic stroke, is required to improve recovery and brain repair process after stroke^29,30^.

### Limitation of study

This study did not have the same blood collection time, and this study also did not compare each stroke risk factor in every subject.

## Conclusion

There was a relationship between the time of collection of blood and the level of EPO and also there was difference EPO level in lacunar stroke subtype compared with non-lacunar. Future research using prospective method is needed regarding EPO level and clinical outcome in acute ischemic stroke comparing each stroke risk factor and the same blood collection time.

### Supplementary Information

Supplementary Information.

## Data Availability

The datasets used and/or analysed during the current study available from the corresponding author on reasonable request.
