# Supplementary material for: Serum erythropoietin in acute ischemic stroke: preliminary findings
Source: Sci Rep. 2024 Feb 1;14:2661. doi: 10.1038/s41598-024-53180-3 (PMC10834471; doi:10.1038/s41598-024-53180-3)
Supplement: Supplementary file 1 — Supplementary Information. [file 41598_2024_53180_MOESM1_ESM.pdf]

SUBJECTS RESEARCH DATA

EPO	Age	HB	Ht	Leukosit	Eritrosit	Trombosit	RBG	SGOT	SGPT
4104.4	68	14.6	42.9	7.37	5.1	259000	171		
980	62	14.8	43.1	9670	5.05	283000	297		
705.5	46	15.7	44.2	11090	5.39	347000	160		
3048.5									
6267.3	59	13.7	41.6	12920	4.48	323000	112		
4702.2	48	12.7	38.5	8870	4.95	446000	332		
855.8	61	14.9	45.3	9130	5.19	273000	137	30	21
30500	69	16.3	48.2	7380	5.63	248000	171		
5079.9	62	14.6	42.9	861	507	237000	243		
837.3	49	15.4	45.9	8370		375000	68		
856.7	69	16.3	48.2	7380	5	248000	171		
887.7	58	16.4	48.5	6400	5.45	329000	90	18	41
6118.6	48	15.9	47	15630	5.48	383000	90	16	27
870.3	51	13.9	40.2	7390	4.88	207000	156		
4323.5	68	14.2	38.8	10650	4.35	286000	142	20	17
2430.3	55	13.5	39.7	6630	4.92	252000	90		
6286.1	51	13.7	40.2	6050	4.46	253000	93		
2988.9	70	12.8	36.6	15000	4.28	206000	116		
973.8	45	15.1	42.5	13440	5.11	326000	133	33	50
998.2	60	14.1	43.6	11000	5.26	307000	156		
2441.8	70	12.8	36.6	15000	4.28	206000	116		
482.3	56	11.7	36.2	8910	4.11	326000	132		
2797.5	51	16.5	50	9400		327000	136		
463.3	65	15	44.6	9050	5.45	257000	164		
431	55	13.9	40.5	10020		280000	126		
134.2	70	12.8	36.6	15000	4.28	206000	116		
427	45	15.6	46.9	9300	5.53	239000	142		
202.9	70	12.8	36.6	15000	4.28	206000	116		
992.8	58	16.4	48.5	6400	5.45	329000	90	18	41
1180	48	15.9	47	15630	5.48	383000	90	16	27
536.9	51	13.9	40.2	7390	4.88	207000	156		
820.6	43	10.5	33.5	15.8	4.19	538000	92	23	31
501.3	52	14.3	44.3	9930	4.85	261000	101		
434.6	51	14.5	43.1	11740	5.59	238000	205		
258	60	15.8	47.8	7090	5.81	255000	136		
452.9	70	12.8	36.6	15000	4.28	206000	116		
159.9	59	15.9	47.5	8460	5.36	261000			
1205.1	68	14.6	42.9	7.37	5.1	259000	171		
295.3	62	14.8	43.1	9670	5.05	283000	297		
993	46	15.7	44.2	11090	5.39	347000	160		
847.4									
480.7	59	13.7	41.6	12920	4.48	323000	112		
784.4	48	12.7	38.5	8870	4.95	446000	332		
1105.8	61	14.9	45.3	9130	5.19	273000	137	30	21

7766.6	69	16.3	48.2	7380	5.63	248000	171		
173.1	62	14.6	42.9	861	507	237000	243		
808.5	49	15.4	45.9	8370		375000	68		
957.6	69	16.3	48.2	7380	5	248000	171		
755.7	58	16.4	48.5	6400	5.45	329000	90	18	41
710.3	48	15.9	47	15630	5.48	383000	90	16	27
836.5	51	13.9	40.2	7390	4.88	207000	156		
193.4	68	14.2	38.8	10650	4.35	286000	142	20	17
469.4	55	13.5	39.7	6630	4.92	252000	90		
868.9	51	13.7	40.2	6050	4.46	253000	93		
858.8	70	12.8	36.6	15000	4.28	206000	116		
175.7	45	15.1	42.5	13440	5.11	326000	133	33	50
239.5	60	14.1	43.6	11000	5.26	307000	156		

Ur	Cr	Na	K	Ca	Mg	Total chol	HDL	LDL	TG
34	1.41	140	4.3	5.09	2	189	45	137	90
19	0.73	139	4.8			190	40	123	205
23	0.86	139	4.1	5.33	2.3	215	38	176	122
44	1.73	143	4.6	5.37	2.2	186	40	119	186
11	0.77	140	4.5	5.73		220	37	157	216
23	0.77	142	4.8	4.57		386	43	146	459
22	1.01	145	3.8						
25	1.03	139	4.3	4.77	2.1	186	53	125	85
21	1.07	145	3.9	3.9	2.4	163	34	116	76
22	1.01	145	3.8						
42	0.91	140	4	4.05	2.1	214	43	161	87
20	1.03	144	3.5			223	43	161	192
30	0.85	142	3.6	5.53	1.6	215	45	151	87
20	0.85	144	3.6	4.97		196	35	143	132
30	0.84	142	4.1	5.05	2	145	44	90	109
23	0.63	145	3.8			205	65	131	46
27	0.92	141	3.6	4.65	2.3	175	37	121	103
14	0.7	140	3.6	4.05		212	40	162	135
17	0.74	140	3.8			235	42	173	121
27	0.92	141	3.6	4.65	2.3	175	37	121	103
26	0.91	140	3.8	4.25	2.3	160	37	108	86
24	0.8	138	4.1	4.29	2	221	41	149	230
19	0.78	141	3.4	4.29		236	63	154	105
22	0.76	139	3.5			211	59	118	148
27	0.92	141	3.6	4.65	2.3	175	37	121	103
142	1.21		3.4			217	58		123
27	0.92	141	3.6	4.65	2.3	175	37	121	103
42	0.91	140	4	4.05	2.1	214	43	161	87
20	1.03	144	3.5			223	43	161	192
30	0.85	142	3.6	5.53	1.6	215	45	151	87
34	0.71	138	4.5	5.25	2.1	172	24	113	206
41	1.35	140	3.8			160	41	111	73
19.7	0.9					280	54	210	73
21	1.43	142	3.5			186	40	129	115
27	0.92	141	3.6	4.65	2.3	175	37	121	103
38	1.18	137	4.3			297	42	203	148
34	1.41	140	4.3	5.09	2	189	45	137	90
19	0.73	139	4.8			190	40	123	205
23	0.86	139	4.1	5.33	2.3	215	38	176	122
44	1.73	143	4.6	5.37	2.2	186	40	119	186
11	0.77	140	4.5	5.73		220	37	157	216
23	0.77	142	4.8	4.57		386	43	146	459

22	1.01	145	3.8						
25	1.03	139	4.3	4.77	2.1	186	53	125	85
21	1.07	145	3.9	3.9	2.4	163	34	116	76
22	1.01	145	3.8						
42	0.91	140	4	4.05	2.1	214	43	161	87
20	1.03	144	3.5			223	43	161	192
30	0.85	142	3.6	5.53	1.6	215	45	151	87
20	0.85	144	3.6	4.97		196	35	143	132
30	0.84	142	4.1	5.05	2	145	44	90	109
23	0.63	145	3.8			205	65	131	46
27	0.92	141	3.6	4.65	2.3	175	37	121	103
14	0.7	140	3.6	4.05		212	40	162	135
17	0.74	140	3.8			235	42	173	121

FBG	2jppBG	Ur acid	HbA1C
143		5.1	
242		2.6	
120		5.5	
141	131	5.8	
218		4.3	10.1
148	183	4.8	7.3
201	362	5.7	9
82	96	4.8	5.1
96	3.4	6.5	
104	98	6.2	6.1
188	184	6.3	6.4
115	166	4.9	
		6.8	
87		7.8	
93		5.1	
121			
166	179	4.5	6.6
93		5.1	
116		5.1	
116		6.3	
150		3	
108	132	3.3	5.1
93		5.1	
102		7.4	5.4
93		5.1	
96	3.4	6.5	
104	98	6.2	6.1
188	184	6.3	6.4
91	109	5.6	
109		13.9	102
218	281	4.4	
146	92	6.2	
93		5.1	
146	135	7.7	
143		5.1	
242		2.6	
120		5.5	
141	131	5.8	
218		4.3	10.1
148	183	4.8	7.3

201	362	5.7	9
82	96	4.8	5.1
96	3.4	6.5	
104	98	6.2	6.1
188	184	6.3	6.4
115	166	4.9	
		6.8	
87		7.8	
93		5.1	
121			
166	179	4.5	6.6
